# Team Size and Stretching-Exercise Effects on Simulated Chest Compression Performance and Exertion

**DOI:** 10.5811/westjem.2017.8.34236

**Published:** 2017-09-11

**Authors:** Jessica C. Schoen, Jason T. Machan, Max Dannecker, Leo Kobayashi

**Affiliations:** * Alpert Medical School of Brown University, Department of Emergency Medicine, Providence, Rhode Island; † Lifespan Medical Simulation Center, Providence, Rhode Island; ‡ Mayo Clinic Rochester, Department of Emergency Medicine, Rochester, Minnesota; § Rhode Island Hospital, Biostatistics Core, Providence, Rhode Island

## Abstract

**Introduction:**

Investigators conducted a prospective experimental study to evaluate the effect of team size and recovery exercises on individual providers’ compression quality and exertion. Investigators hypothesized that 1) larger teams would perform higher quality compressions with less exertion per provider when compared to smaller teams; and 2) brief stretching and breathing exercises during rest periods would sustain compressor performance and mitigate fatigue.

**Methods:**

In Phase I, a volunteer cohort of pre-clinical medical students performed four minutes of continuous compressions on a Resusci-Anne manikin to gauge the spectrum of compressor performance in the subject population. Compression rate, depth, and chest recoil were measured. In Phase II, the highest-performing Phase I subjects were placed into 2-, 3-, and/or 4-compressor teams; 2-compressor teams were assigned either to control group (no recovery exercises) or intervention group (recovery exercises during rest). All Phase II teams participated in 20-minute simulations with compressor rotation every two minutes. Investigators recorded compression quality and real-time heart rate data, and calculated caloric expenditure from contact heart rate monitor measurements using validated physiologic formulas.

**Results:**

Phase I subjects delivered compressions that were 24.9% (IQR1–3: [0.5%–74.1%]) correct with a median rate of 112.0 (IQR1–3: [103.5–124.9]) compressions per minute and depth of 47.2 (IQR1–3: [35.7–55.2]) mm. In their first rotations, all Phase II subjects delivered compressions of similar quality and correctness (p=0.09). Bivariate analyses of 2-, 3-, and 4-compressor teams’ subject compression characteristics by subsequent rotation did not identify significant differences within or across teams. On multivariate analyses, only subjects in 2-compressor teams exhibited significantly lower compression rates (control subjects; p<0.01), diminished chest release (intervention subjects; p=0.03), and greater exertion over successive rotations (both control [p≤0.03] and intervention [p≤0.02] subjects).

**Conclusion:**

During simulated resuscitations, 2-compressor teams exhibited increased levels of exertion relative to 3- and 4-compressor teams for comparable compression delivery. Stretching and breathing exercises intended to assist with compressor recovery exhibited mixed effects on compression performance and subject exertion.

## INTRODUCTION

Effective chest compressions are paramount for successful resuscitation of cardiac arrest.^1^ Compressions of adequate rate and depth and reduced “hands-off time” maximize coronary perfusion pressure and improve the likelihood of return of spontaneous circulation.^1^–^5^ However, the quality of chest compressions performed *in situ* during inhospital and out-of-hospital cardiac arrest resuscitations continues to be poor.^1^,^6^,^7^

Provider fatigue is a major factor in the quality of chest compressions; several studies have demonstrated a significant decay in compression quality after 60 to 90 seconds of continuous compressions.^8^–^13^ Studies evaluating groups of providers performing continuous compressions in intervals separated by periods of rest^8^,^14^ demonstrated that the quality of compressions improved in the first minute of an individual compressor’s rotation when compared with the last minute of the same compressor’s preceding rotation, suggesting some degree of recovery during rest.^8^,^14^ This recovery pattern was observed for single providers with short rest (30 seconds)^8^ as well as for teams of rotating providers with longer rest (1–3 minutes).^14^

Along with increasing the size of provider teams performing compressions so as to prolong rest periods, another approach to mitigate provider fatigue is to actively facilitate recovery during rest periods. Static stretching of muscles in lengthened positions for a prescribed period of time is commonly practiced before exercise alone or as part of a warm-up routine.^15^,^16^ Some studies suggest improved performance following static stretching,^15^,^17^ while others suggest that stretching can reduce maximal muscle performance (although minimally).^15^,^16^ Additionally, deep breathing exercises are commonly used relaxation techniques,^18^ and there is evidence to suggest that even a short duration (two minutes) deep-breathing exercise improves lung function, heart rate, and blood pressure.^19^–^20^

Given the variety of factors contributing to chest compressor exertion and potential approaches to mitigate compressor fatigue, we set out to study the impact of 1) the size of resuscitation teams, specifically the number of alternating providers delivering chest compressions; and 2) targeted rest-recovery exercises on chest compression quality and individual provider exertion during a simulated cardiac arrest scenario. The primary hypothesis was that larger teams of compressors would deliver sustained high-quality compressions with less individual-provider exertion compared to smaller teams. The secondary hypothesis was that rest periods with an experimental recovery exercise intervention between compression rotations would mitigate provider fatigue and help maintain high-quality chest compressions throughout the resuscitation. Although previous investigations have independently evaluated resuscitation team size, rest duration, and recovery on chest compression performance and fatigue,^14^,^21^ this study was the first to concurrently evaluate their effects on compression quality and individual providers’ levels of exertion.

**Population Health Research Capsule**

What do we already know about this issue?

Effective chest compressions are critical for resuscitation success, but difficult to perform. Provider fatigue is a limiting factor; mitigating fatigue may improve compression quality.

What was the research question?

Do larger compression team sizes or targeted recovery exercises improve compression quality and reduce provider exertion?

What was the major finding of the study?

Smaller teams exhibited greater exertion than larger teams. Recovery exercises had mixed effects on performance and exertion.

How does this improve population health?

Implementing strategies to mitigate provider fatigue may facilitate sustained high quality chest compressions and may improve the likelihood of successful resuscitation of cardiac arrest.

## METHODS

### Study Design, Setting, and Population

The prospective simulation study was approved by the institutional review board. Investigators conducted the study in two phases, with Phase I assessing and ranking volunteering subjects by quality of chest compressions, and Phase II testing study hypotheses on the highest-ranked Phase I subjects. Phase I of the study was conducted on campus at a medical school facility, and Phase II was conducted at a hospital-affiliated medical simulation center. First- and second-year medical students were recruited via email and voluntarily enrolled in the study sessions. The study was conducted from February 2015 to June 2015.

A convenience sample of 45 medical students was used for the study. Defined and limited by the research budget, the study sample size was comparable to those of similar studies that previously evaluated chest compression quality and provider fatigue in simulated settings.^8^–^12^,^14^,^21^,^22^

### Phase I Study Protocol, Metrics, and Sessions

Investigators obtained written informed consent and self-reported demographic data from all participants (gender, age, height, weight, previous cardiopulmonary resuscitation [CPR], basic life support [BLS] or advanced cardiovascular life support [ACLS] training, and year in medical school). After a scripted introduction to research aims, study methods and simulation setting, subjects were oriented to the study manikin system. A Resusci-Anne SkillReporter manikin (Laerdal, Wappingers Falls, NY) was situated on a waist-height table, a footstool was provided, and compressions were performed in the standing position. Immediately prior to data collection, participants practiced chest compressions by performing approximately 20 compressions with real-time performance feedback from PC SkillReporting software v2.1.0.

After orientation, each subject independently performed four minutes of continuous chest compressions on the manikin without real-time feedback. Continuous measurements of compression rate, compression depth, and chest recoil were obtained. At the end of the Phase I session, each participant received a $10 gift card. Study-subject performance metrics were composited into a ranking list based on each individual participant’s total number of correct compressions delivered.

### Phase II Study Protocol, Metrics, and Sessions

#### Subject Assignment to Resuscitation Teams of Different Sizes

The best-performing Phase I participants were offered the opportunity to enroll in the study’s second phase. Based on an anticipated Phase II enrollment of 30 Phase I subjects (top 75% of Phase I performers), investigators planned to assign volunteering Phase II subjects to fifteen 2-compressor, ten 3-compressor, and/or six 4-compressor teams. Each enrolled Phase II participant completed a minimum of two distinct sessions in different teams that were scheduled at least three hours apart to ensure adequate subject recovery. Each individual Phase II subject received an additional $25 gift card for continued participation.

#### Chest Compression Quality Metrics

**Figure 1:**
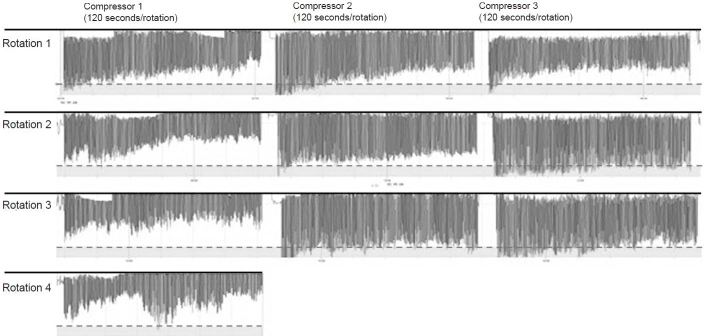
Composite screen-capture image of chest compression dataset visualization for a 3-compressor team’s 20-minute study session, starting at left with time progression to the right and downward. Compression plots for each member of the team are displayed in columns; compression plots for each 3-compressor rotation are displayed in rows. Note the generally inadequate compression depth (appropriate depth indicated by dashed bars), frequency of inadequate chest release (leaning), progressive reduction in compression depth within each compressor’s rotation, as well as the between-compressor and within-compressor variability in compression performance.

Each Phase II team performed continuous chest compressions for a 20-minute simulated cardiac arrest resuscitation with sequential compressor rotation every two minutes in accordance with 2010 American Heart Association guidelines,^2^ e.g.*,* subject 1 (two minutes), then subject 2 (two minutes), then subject 3 (two minutes), then subject 1 (two minutes), etc., for a 3-compressor team. Simulation time was strictly monitored with a stopwatch, and participants were notified at the one minute, one minute forty-five second, and two minute marks; 10 seconds were allotted for switching between team members and for facilitation of subsequent review of individual compressor performances. Measurements of compression rate, compression depth, and chest recoil were continuously obtained (Figure 1). Of note, Phase II compressions were performed by kneeling subjects on a manikin placed on the floor in order to accommodate the significant height differences between alternating subjects, as the frequent bed-height adjustments necessary to minimize subject height-related research confounds otherwise would have proved impractical. (Previous studies have evaluated the quality of continuous chest compressions while kneeling, standing on the floor, or standing on a footstool with bed height adjustment for each provider – these found no significant difference in compression quality between subjects in kneeling and those in footstool positions.^23^,^24^)

Investigators used compressions delivered during a single rotation by an individual subject in 2-compressor (control or intervention group), 3-compressor, and/or 4-compressor teams to calculate the following variables: compression rate, compression depth, and chest recoil; percent of compressions with correct depth and recoil; and number of correct compressions delivered. Each individual subject’s changes (Δ) in performance metrics across his/her compressor rotations were calculated for each rotation relative to his/her first rotation performance.

#### Exertion Metrics

In order to measure compressor exertion, all subjects wore chest-strap heart rate monitors (H7, Polar Electro, Kempele, Finland) paired wirelessly with Polar Beat iOS software v1.4.4 on iPod Touch 5 devices (Apple, Cupertino, CA) to acquire real-time heart rate (HR) data during their compression rotations. Investigators used HR and demographic data to derive the following surrogate metrics for each subject’s level of exertion: 1) percent attained of his/her maximal predicted heart rate, i.e.*,* %mHR = [mean HR during a compression rotation] / [predicted maximal HR derived with the Tanaka formula^25^]; and 2) calculated estimate of caloric expenditure (in kilocalories [kcal]) during a compression rotation.^26^

#### Recovery Intervention

**Figure 2:**
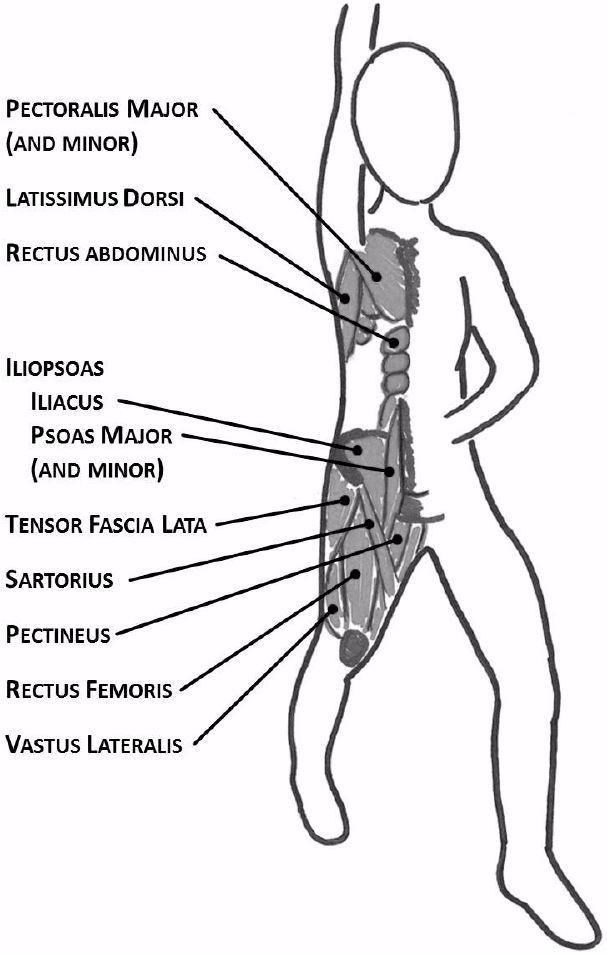
Diagram demonstrating recovery intervention with stretching and concurrent diaphragmatic breathing for experimental 2-compressor teams. The stretch was a shallow lunge with ipsilateral arm raised in the air; the highlighted muscle groups are those targeted by the stretch. Participants were instructed to switch sides approximately every 30 seconds.

To address the secondary research objective, a physical therapist was consulted to assist with the development and implementation of an efficient stretching and breathing exercise that could be easily performed by compressors during their rest periods. The intervention consisted of a stretch and concurrent diaphragmatic breathing – the stretch was a shallow lunge with ipsilateral arm raised in the air (Figure 2); participants were instructed to switch sides approximately every 30 seconds. Only the 2-compressor teams were assigned to one of seven control or six intervention groups prior to the start of the session (due to inadequate numbers of 3- and 4-compressor teams for controlled experimentation). Those in the intervention group were instructed on how to perform the stretch and diaphragmatic breathing exercises immediately before simulations; control subjects were instructed to rest in a chair between compressor rotations.

#### Data Analysis

Investigators analyzed participant demographic data on age, gender, height, weight, and body mass index (BMI) with Kruskal-Wallis and Fisher’s exact tests. Subject compression performance data were summatively characterized by compression rate, compression depth and chest recoil; percent of compressions with correct depth and recoil; and number of correct compressions delivered. Each subject’s HR and caloric expenditure data were managed as continuous variables and analyzed across compressor rotations by assignment to control or intervention group and by team size. All compression and exertion metrics were modeled using binominal generalized linear mixed models, nesting observations within each subject and with a pre-specified α level of 0.05 (SAS version 9.3; SAS Institute, Cary, NC). Team size, intervention, and compression rotation were treated as fixed effects along with all interactions, with the three-way interaction treated as the primary hypothesis test for differential change according to study group.

## RESULTS

### Phase I Results

Of 49 subjects who expressed interest in the study, 45 enrolled and six canceled due to schedule conflicts for a total of 39 participants in Phase I. Complete Phase I data were available for 36 subjects (three subjects’ data were lost to equipment malfunction). Subjects were a median of 24.0 (interquartile range [IQR]1–3: [23.0–26.0]) years of age with a median BMI of 23.7 (IQR1–3: [22.6–24.5]); 11 (27.8%) subjects were female. All subjects had received BLS training within the prior two years. Phase I chest compressions exhibited a median rate of 112.0 (IQR1–3: 103.5–124.9) compressions per minute (cpm), median depth of 47.2 (IQR1–3: [35.7–55.2]) mm, and were 24.9% (IQR1–3: [0.5%–74.1%]) correct.

### Phase II Results

**Figure 3:**
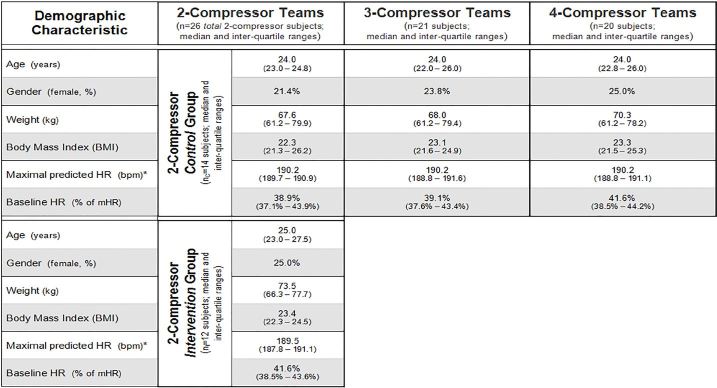
Subject demographic characteristics: There were no significant differences in reported and calculated values for all study teams (Kruskal-Wallis test for continuous variables and Fisher’s exact [2×4] test for binary variables). *bpm*, beats per minute; *HR*, heart rate; *kg*, kilogram; *mHR*, maximal predicted HR.

Twenty-six Phase II subjects comprised thirteen 2-compressor teams (seven control and six intervention teams); seven 3-compressor teams; and five 4-compressor teams. Demographic characteristics of the control and intervention 2-compressor teams, the 3-compressor teams, and 4-compressor teams were similar (Figure 3). Phase II subjects’ median BMI was 23.0 (IQR1–3: [21.5–24.8]), and their median baseline HR was 40.7% (IQR1–3: [37.3%–43.9%]) of their predicted maximal HR.

#### Effects of Resuscitation Team Size

##### Chest Compression Quality

**Figure 4:**
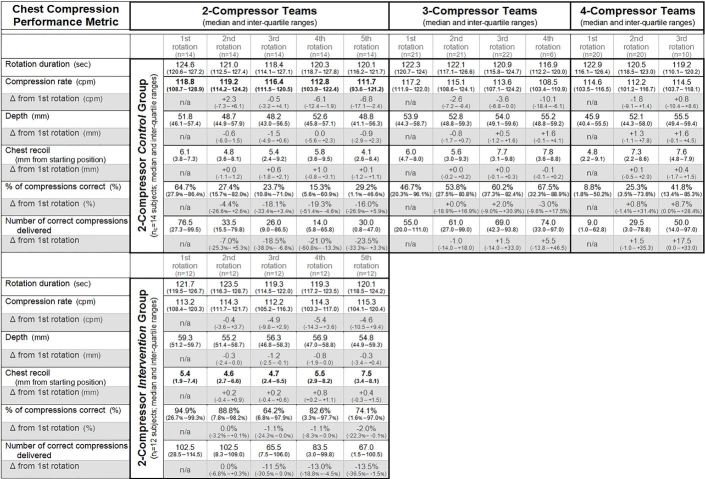
Subject compression performance across rotations by teams Results of descriptive statistics and comparative analyses of compressions by 2- (control and intervention), 3-, and 4-compressor teams are presented in tabular groups. Significant differences on multivariate analyses by study group (team size; control or intervention) and rotation are in bold. *cpm*, compressions per minute; *HR*, heart rate; *kcal*, kilocalories; *mHR*, maximal predicted HR; *mm*, millimeter; *sec*, second.

During their first compressor rotations, subjects in 2- (control and intervention), 3-, and 4-compressor teams did not exhibit differences in their compression rates (p=0.34), depths (p=0.25), and recoil (p=0.82); percent and number of correct first-rotation compressions were not significantly different between all study groups (p=0.09); see Figure 4 for details. Multivariate analyses by team size, intervention and compressor rotation number revealed that 2-compressor control team subjects delivered fewer compressions in later rotations (mean estimated slope of change over each subject’s sequential compressor rotations, with confidence intervals [CI]: −3.4% [95% CI {−5.7% to −1.3%}] in cpm per rotation; p<0.01). The number of correct compressions, proportion of correct compressions, and compression depth were not different across different teams or rotations on multivariate analyses.

##### Exertion

**Figure 5:**
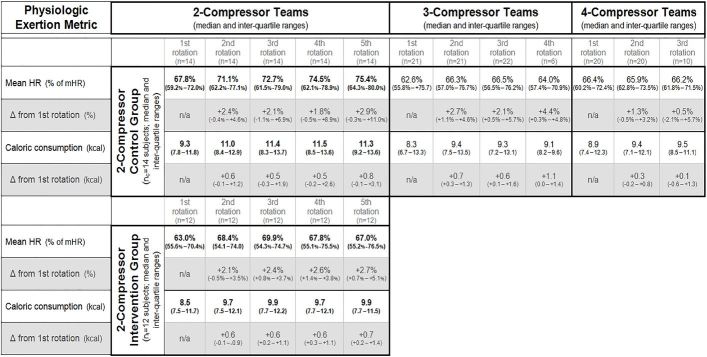
Subject exertion across rotations by teams. Results of descriptive statistics and comparative analyses of individual exertion in 2- (control and intervention), 3-, and 4-compressor teams are presented in tabular groups. Significant differences on multi-variate analyses by study group (team size; control or intervention) and rotation are in bold; see text for details. *cpm*, compressions per minute; *HR*, heart rate; *kcal*, kilocalories; *mHR*, maximal predicted HR; *mm*, millimeter; *sec*, second.

At baseline, all study groups had similar median %mHR (p=0.73) and caloric expenditure (p=0.85); see Figure 5 for details. Subjects in the 2-compressor teams (both control and intervention) exhibited significant increases in exertion (and energy expenditure) over successive compressor rotations; see below for additional details by specific study group assignment. Changes in exertion (and energy expenditure) over successive rotations for subjects in 3-compressor teams and 4-compressor teams did not attain significance.

#### Effects of Recovery Intervention

##### Chest Compression Quality

Comparison of the 2-compressor control group with the 2-compressor intervention group revealed two significant differences in chest compression quality. Unlike the 2-compressor intervention subjects, the 2-compressor control subjects exhibited diminishing compression rates over successive compression rotations. Additionally, the 2-compressor intervention subjects exhibited increased leaning in later rotations, with a mean estimated slope of change over each subject’s sequential compressor rotations of +0.2 [95% CI {+0.1 to +0.4}] mm per rotation (p=0.03) when compared against 2-compressor control group subjects who exhibited no significant change in leaning across rotations.

##### Exertion

Subjects in both the 2-compressor control group and 2-compressor intervention group exhibited increasing levels of exertion and energy expenditure over successive rotations. The 2-compressor control subjects who did not perform the experimental recovery intervention exhibited the following changes: +3.0 [95% CI {+0.9 to +5.2}] %mHR per rotation (p=0.02) and +0.4 [95% CI {+0.1 to +0.8}] kcal per rotation (p=0.03). The 2-compressor intervention subjects who performed experimental stretching and breathing exercises exhibited the following changes: +1.4 [95% CI {+0.6 to +2.3}] %mHR per rotation (p=0.01) and +0.2 [95% CI {+0.1 to +0.3}] kcal per rotation (p=0.02).

## DISCUSSION

The 2010 AHA guidelines recommended rotating providers performing chest compressions every two minutes to mitigate provider fatigue.^2^ Several previous investigations have evaluated the effect of rest duration on chest compression quality and fatigue.^8^,^14^ In large healthcare facilities, cardiac arrest resuscitation teams can potentially include three, four, or more compressors who each have several minutes of rest between compression sets. Given that a significant proportion of chest compressions and cardiac resuscitations take place in settings with smaller cohorts of qualified personnel on duty at any one time, investigators applied basic cardiac arrest resuscitation simulation scenarios to examine the potential impact of smaller team sizes on compression performance and compressor exertion.

The experimental study of 2-, 3-, and 4-compressor teams revealed a definite reduction of approximately 3% in compression rate per rotation for only control 2-compressor teams on multivariate analyses (and without changes in other compression characteristics). Concurrently, levels of subject exertion displayed small, orderly, and coherent differences that were significantly associated with study group assignment. Specifically, all subjects featured similar baseline %mHR measurements despite considerable differences in the quality of their chest compressions. It is therefore of interest to note that subsequent physiologic monitoring revealed that only subjects in the 2-compressor teams exhibited increases in caloric expenditure and exertion over successive rotations. These findings suggest that the size of a smaller CPR compressor team may have a demonstrable effect on the quality of chest compressions delivered and provider exertion over the course of a typical cardiac arrest resuscitation. At the same time, the data suggest that teams with more than three compressors do not appear to differentially perform higher-quality chest compressions or exhibit reduced levels of provider exertion. Larger teams may therefore elect to direct additional personnel resources to perform other critical tasks during resuscitations without potentially compromising the quality of chest compressions performed.

Investigators also evaluated whether an experimental rest-recovery intervention would reduce provider exertion and improve compression quality in 2-compressor teams. Whereas data regarding the effects of static stretching on exercise performance are conflicting,^15^–^17^ several previous studies have demonstrated improvement in pulmonary and cardiovascular function with deep breathing exercises.^19^,^20^ In the current study, the 2-compressor team subjects who performed the recovery intervention exercises during rest periods were able to sustain adequate chest compression rates throughout the simulation and with less exertion when compared to control group 2-compressor team subjects. This suggests that targeted recovery exercises performed during rest periods may help mitigate provider fatigue and help facilitate the sustainment of adequate chest compression quality during cardiac arrest resuscitations. Providers in clinical practice settings where few compressors are available may benefit from performing the studied recovery exercises during cardiac arrest resuscitations.

This study’s findings are in agreement with numerous previous studies that have established the provider-dependent and generally poor quality of CPR.^1^,^6^,^7^,^22^,^27^ Study simulation sessions elicited a broad spectrum of chest compression performances from a cohort of young medical student subjects with normal BMI. Objective measurements revealed that 25% of Phase 1 participants were unable to deliver a single correct compression during their four-minute simulation session (data not shown). Despite the use of Phase I sessions as a screening process to enroll higher-performing subjects, approximately 50% of all Phase II chest compressions were still performed incorrectly (primarily due to inadequate compression depth). These findings raise significant concerns with respect to the current approaches of training and entrusting the delivery of critical life-saving interventions to the general CPR resuscitator cohort. Furthermore, specific subject predictors of chest compression performance could not be derived from the dataset, with only correlations of intermediate strength identified between compression quality and subject weight (r=0.4) or BMI (r=0.35) for the studied demographic characteristics (data not shown).

It is somewhat remarkable that a significant proportion of the study’s healthy, young, motivated, and recently-trained medical students delivered suboptimal (simulated) chest compressions. Accordingly, we expect that a typical CPR team (comprised of individuals of greater diversity with respect to age, gender, and baseline health and physical capability) would deliver chest compressions of even poorer quality in simulated and/or live settings. As a result, next-step interventions are not entirely clear. Advocacy for more frequent training with the judicious use of simulation technologies and the widespread application of accelerometer-based, real-time feedback devices for monitoring and assurance of compression quality are distinct possibilities.^1^,^28^

On the other hand, the objective identification and in-resuscitation recognition of poor CPR quality may fail to resolve a critical issue, i.e., the existence of a significant population of up-to-date BLS-certified healthcare professionals who are physically incapable of delivering chest compressions as specified by formal guidelines. The challenge is further complicated by the failure of mechanical auto-compression devices (engineered to methodically deliver correct compressions) to improve patient outcomes.^29^ In the interim, further investigation into compressor team compositions and rest-recovery interventions may be warranted.

## LIMITATIONS

The research budget and subject pool limited the Phase I sample size; investigators were unable to enroll a sufficient number of Phase II subjects to reach the target number of study teams. Study groups of 2-, 3-, and 4-compressor teams may not have been fully matched in baseline performance; this limited comparative assessments across teams of different sizes.

## CONCLUSION

Members of 2-compressor teams exhibited greater levels of exertion relative to members of larger compressor teams for comparable simulated chest compression performance. Stretching and breathing exercises intended to assist with compressor recovery exhibited mixed effects on compression performance and subject exertion.
